# Accurate discharge and water level forecasting using ensemble learning with genetic algorithm and singular spectrum analysis-based denoising

**DOI:** 10.1038/s41598-022-22057-8

**Published:** 2022-11-18

**Authors:** Anh Duy Nguyen, Phi Le Nguyen, Viet Hung Vu, Quoc Viet Pham, Viet Huy Nguyen, Minh Hieu Nguyen, Thanh Hung Nguyen, Kien Nguyen

**Affiliations:** 1 School of Information and Communication Technology, Hanoi University of Science and Technology, Hanoi, Vietnam; 2 Institute for Advanced Academic Research, Chiba University, Chiba, Japan

**Keywords:** Computer science, Hydrology

## Abstract

Forecasting discharge (Q) and water level (H) are essential factors in hydrological research and flood prediction. In recent years, deep learning has emerged as a viable technique for capturing the non-linear relationship of historical data to generate highly accurate prediction results. Despite the success in various domains, applying deep learning in Q and H prediction is hampered by three critical issues: a shortage of training data, the occurrence of noise in the collected data, and the difficulty in adjusting the model’s hyper-parameters. This work proposes a novel deep learning-based Q–H prediction model that overcomes all the shortcomings encountered by existing approaches. Specifically, to address data scarcity and increase prediction accuracy, we design an ensemble learning architecture that takes advantage of multiple deep learning techniques. Furthermore, we leverage the Singular-Spectrum Analysis (SSA) to remove noise and outliers from the original data. Besides, we exploit the Genetic Algorithm (GA) to propose a novel mechanism that can automatically determine the prediction model’s optimal hyper-parameters. We conducted extensive experiments on two datasets collected from Vietnam’s Red and Dakbla rivers. The results show that our proposed solution outperforms current techniques across a wide range of metrics, including NSE, MSE, MAE, and MAPE. Specifically, by exploiting the ensemble learning technique, we can improve the NSE by at least $2\%$. Moreover, with the aid of the SSA-based data preprocessing technique, the NSE is further enhanced by more than $5\%$. Finally, thanks to GA-based optimization, our proposed model increases the NSE by at least $6\%$ and up to $40\%$ in the best case.

## Introduction

Rivers are essential for crop irrigation and the survival of life. River flow fluctuations have a wide range of effects on the climate and ecology. The extreme rise or drop in the volume or level of water in the rivers produces significant floods or droughts. Both kinds of disasters would result in severe losses. According to the World Health Organization (WHO)^1^, more than 2 billion people were impacted by floods between 1998 and 2017, and it is anticipated that 700 million people will be in danger of being displaced by drought by 2030. In addition, according to this organization, eighty to ninety percent of natural disasters in the last ten years are from floods, droughts, and severe storms. Being aware of that fact, predicting future discharges (Q) and water levels (H) is of great importance because it is indispensable in dealing with various problems, including disaster forecasting (potentially flood, drought), water resources distribution (for irrigation), water reserving for dry seasons, and maintaining healthy freshwater for human sanitization.

Numerous efforts have been made to predict (Q) and (Q) values in the literature, which can be divided into two main categories: process, physical-based numerical models, and data-driven models. The methods belonging to the former type share some common characteristics: they usually require hardly measured input data, mostly based on the study of atmospheric circulation, the evolution of long-term weather processes, and physical conditions^2^. These models can extract the intrinsic behaviors of hydrological variables by conceptualizing the physical processes and features. However,these approaches usually require special knowledge and techniques in the field; thus, enclosing the research zone to only hydrological researchers. Another limitation of the physical-based numerical models is that they are usually mathematically heavy, unique dedicated numerical formulas, struggling to estimate huge parameters and expensive computational cost. Moreover, these models are designed differently for specific regions and generally not applicable to others, especially for areas where not all parameters are recorded.

The latter approach is categorized into traditional machine learning models and deep learning models. The traditional machine learning group includes simple models such as Autoregressive family (i.e., the Autoregressive (AR), the Autoregressive Moving Average (ARMA), and the Autoregressive Integrated Moving Average (ARIMA) models^3^), Support Vector Machine (SVM) alternatives^4^, and Random Forest (RF). In spite of their simplicity, the traditional machine learning-based approaches are incapable of capturing the complex correlation between the input data and the desired output; hence, they cannot achieve high prediction accuracy. These trade-offs between the predictive ability of a model and its complexity have been well illustrated in various previous works^14,15^.

Deep learning has recently gained popularity as a powerful technique for extracting intricate non-linear correlations from data and delivering high accuracy in a variety of tasks such as classification and prediction. Several deep learning techniques have been used in prediction tasks including Multilayer Perceptron (MLP or ANN for simplicity)^16^, Long Short-Term Memory (LSTM)^17,18^, and Convolutional Neural Network (CNN)^19,20^.

Despite the outstanding potential of deep learning in producing highly accurate prediction results, the existing deep learning-based discharging and water level prediction encounters three challenges: insufficiency of training data, presence of noise and outliers, and difficulties in optimizing the model’s hyper-parameters. Regarding the first issue, namely the data shortage, it is well-known that data on discharge and water level are difficult to obtain, particularly in developing countries. Furthermore, the data acquired is likely to be missing due to instrument failure or environmental circumstances. These difficulties would result in a training data set lacking generalizability, hence decreasing the model’s accuracy. The second problem is the appearance of noise and outliers in the data. Outliers arise at times of extraordinary weather changes, and noise may be produced due to equipment errors. Noise and outliers cause the model training more challenging and diminish the prediction accuracy. Finally, adjusting model hyper-parameters is a typical challenge when employing deep learning models. Usually, ones will choose parameters based on experience or by trial-and-error. However, these methods either consume lots of time for determining the optimal solution or may not always find a good enough one.

Motivated by the aforementioned findings, we propose in this paper a novel method for reliably predicting the Q and H values that overcomes all of the limitations of previous studies. For the first problem, we use the ensemble learning technique to compensate for the shortage of training data. Our ensemble model, in particular, consists of several base learners and one meta learner. The same model structure will be used to train base learners with various hyper-parameters. This mechanism will enable each base learner to learn a new data feature. The trained base learners are then utilized to produce training data for the meta learner. In this way, we can enrich the training data, thus enhance the data’s generality. To address the second problem of noise and outliers, we provide a data preprocessing approach based on the SSA technique. By using SSA, we can decompose the original data into several components, each reflecting a data feature. We then classify the decomposed components into two groups. The former includes the components representing the meaningful data, such as data trend and periodic, while the latter contains the noise-like components. The trends and periodic then will be used to train the prediction model, while the noise will be eliminated. To deal with the last issue relating to hyper-parameter selection, we propose a GA-based algorithm that automatically determines the optimal parameters for the prediction model. GA, a meta-heuristic algorithm inspired by Darwin’s theory of evolution, has been widely used to handle large space search problems. Previous studies have shown that GA can reduce the time complexity while assuring the goodness of the solution.

The following are our paper’s main contributions.

- We propose an ensemble learning technique to address the data scarcity issue in Q and H predictions. Our ensemble model is comprised of multiple base learners and, a meta learner. Each base learner is made up of numerous 1D-CNN and LSTM layers to extract both the short-term and long-term temporal correlation simultaneously. The base learner will be trained with different hyper-parameters and used to generate training data for the meta learner model. The meta learner is composed of two LSTM layers. The first layer is in charge of extracting information from historical data, while the second layer is responsible for retrieving the knowledge acquired by the base learner.
- We leverage SSA to propose a data preprocessing method dealing with noise and outliers. Specifically, we decompose the original data into several components and then apply our proposed algorithm to filter out the significant components and automatically remove the noise.
- We offer a unique GA-based hyper-parameter optimization technique that automatically identifies and trains the prediction model with sub-optimal hyper-parameters.
- We conduct comprehensive experiments on a real dataset gathered from the Hong River in Vietnam to extensively assess and compare the performance of our proposed approach to current methodologies. To the best of our knowledge, this is the first attempt to use deep learning to predict Q and H values in Vietnamese datasets.

As far as we know, we are the first to address the data shortage in dealing with (Q) and (H) predictions. We are also the first to leverage ensemble learning with the aid of GA-based optimization and SSA-based data denoising to enhance the accuracy of the Discharge and Water level prediction problem. In order to demonstrate the efficacy of our approach, we conducted extensive experiments on two real datasets gathered from distinct rivers in Vietnam. Our method surpasses existing benchmarks in the one-step-ahead prediction studies by enhancing the NSE metric from $29.8\%$ to $42.5\%$. A similar pattern is captured in the scenario of multi-step-ahead predictions, where the proposed framework outperforms other models by at least $27\%$ and by $145\%$ in the best case.

The rest of the paper is structured as follows. Section “Related works” briefly introduces some related works. We then present the mathematical formulation of the investigating problem, and the details of our proposed approach in Section “Proposal”. Section “Performance evaluation” evaluates the proposal’s performance and compares it to other benchmarks. Section “Conclusion” concludes the paper and describes our future work.

## Related works

This section focuses on approaches for estimating Q and H values based on machine learning, which can be divided into two groups: traditional machine learning algorithms and deep learning models.

The traditional machine learning group includes simple models such as Autoregressive family, Support Vector Machine (SVM) alternatives^4^, Random Forest (RF). In^5^, the authors presented a comparative study of Random Forests and other statistical methods concerning the lake water level prediction. An Adaptive Metropolis-Markov Chain Monte Carlo-Wavelet Regression (AMMC-MC-WR) model has been proposed to solve the hydrologic time series forecasting in^6^. Hadi G. in^7^ compared the effectiveness of adaptive neuro-fuzzy inference system (ANFIS) and ARIMA models in modeling water level. Chuan Wang et al.^8^ studied the annual runoff forecasting problem. The authors proposed a method that combines ARIMA and ensemble approaches. Authors in^9^, exploited the advantages of both hybrid harmonic analysis and wavelet network in predicting the sea water level. The authors in^10^ combined three models, namely K-means model, support vector classification (SVC) model, and support vector regression (SVR) model, to predict the water level in the context of High Sediment Load Reaches. The authors in^11^ exploited wavelet analysis to decompose the data into low frequency and high frequency components, thereby extract the time-frequency information. The authors then combined wavelet analysis and SVM model to predict lake water level fluctuations. An improvement of the least squares support vector machine (LSSVM) was proposed in^12^ to predict daily water level. In^13^, Seyed et al. trained a model using the radial basis function (RBF) to predict the water level of rivers. The authors leveraged a meta-heuristic algorithm, namely firefly algorithm, to optimize the model’s hyper-parameters. The limitation of the conventional machine learning approach is that it is unable to model the complicated relationships within the data; hence, it is unable to generate highly accurate prediction results.

Recently, deep learning has emerged as an effective method for extracting complex non-linear relationships from data and providing high accuracy in many tasks such as classification or prediction. Several deep learning techniques have been used in prediction tasks including Multilayer perceptron (MLP or ANN for simplicity)^16^, Long Short-Term Memory (LSTM)^17,18^, and Convolutional Neural Network (CNN)^19,20^. Some existing works have combined these techniques to enhance the prediction results. For example, the combination of LSTM and KNN has been exploited in^21^ to predict the flood in China. The authors in^22^ combined ANN, decision tree (DT), random forest (RF), and SVM to forecast the water level in South Korea. In^23^, R. Hu et al. integrated LSTM, proper orthogonal decomposition (POD) and singular value decomposition (SVD) to reduce the dimensional data size. Zhu et al. leveraged MLP and ANN to predict the water level in^16^. The authors in^19^, combined gated recurrent unit (GRU) and CNN to predict the water level in Yangtze River. The authors in^24^ exploited the Extreme Learning Machines approach to predict the daily water level of Urmia Lake. The Extreme Learning Machine is a technique that trains only a single layer. This way allows the model to reduce the training time significantly while maintaining acceptable accuracy. In^18^, the authors exploited LSTM to predict the five-day-ahead discharge in a river in Indonesia. LSTM model was also used in^17^ to predict floods in rivers in Vietnam. Liu et al.^21^ combined LSTM and KNN for real-time flood forecasting in China. The experiment results showed the superiority of LSTM-KNN over LSTM. In^25^, the authors used LSTM with attention mechanism to build a hydrological prediction model for small- and medium-sized rivers. Moreover, they exploited Cuckoo search to determine some important hyper-parameters. Furthermore, spatial and temporal correlations have been combined to enhance the prediction accuracy in^26^. In^19^, the authors used GRU and CNN networks to predict the future water level at eight o’clock of the five day ahead. The model leverages GRU to capture the temporal trends of the water levels data. In the meanwhile, the CNN network is used to learn the spatial correlation of data collected from adjacent stations. T.Ren et al. in^16^ focused on predicting the water level of cascaded channels. The authors proposed a data augmentation method that uses temporal and spatial windows to assemble adjacent time slices, and adjacent channels. Besides, they provided a prediction model that combines MLP network and recurrent network. In^23^, R. Hu et al. proposed a hybrid model that integrates LSTM and POD and SVD to reduce the dimensional data size. In^27^, Karbasi et al. proposed an Auto Encode Decoder Bidirectional Long Short-Term Memory (AED-BiLSTM) model for forecasting weekly Reference evapotranspiration (ETo) of one to three weeks ahead. For training and testing, the authors utilized a twenty-year statistical period. Moreover, the authors proposed a CatBoost-Boruta-based algorithm for identifying the critical lags in the forecasting process. In our previous work^28^, we proposed a deep learning model to predict Q and H values, exploiting CNN, LSTM, and ensemble learning techniques in our previous work. A study in^29^ showed that deep learning outperforms gradient boosting machine in pan evaporation prediction.

Numerous publications have proposed optimizers for controlling input features and determining the optimal hyper-parameters for machine learning models. Allawi et al. investigated the reservoir evaporation prediction problem in^30^. Then, they used a GA to choose suitable input variables. GA is also used in^31^. In this work, the authors proposed combining the Radial Basis Neural Network (RBNN) and GA to solve the problem of river flow prediction. GA is utilized to find the optimal combination of the model’s input that can achieve the highest prediction accuracy compared to all other variants. The authors in^32^ stated that Evolutionary algorithms (EA) could attain sub-optimal solutions for applications with highly non-linear, stochastic, non-differentiable, or discontinuous objective functions. Motivated by this observation, the authors combined several GA algorithms (including GA , particle swarm optimization (PSO) algorithm, and shark machine learning algorithm (SMLA)) with two different forecasting models (including radial basis neural network (RBF-NN) and SVR), to address the reservoir inflow and evaporation prediction problem, In^33^, Yaseen et al. leveraged the complementary strengths of the Bat Algorithm (BA) and PSO algorithm to propose a hybrid optimizer named HB-SA. The hybrid approach is also leveraged in^34^, where the authors integrated Swarm Algorithm (SA) and Grasshopper Optimization Algorithm (GOA). The primary objective is to shine a light on the robust exploratory capability and flexible stochastic nature of GOA, as well as the rapid convergence capability of SA. The authors of^35^ investigated the prediction of monthly runoff in the Mangla watershed in northern Pakistan. As the prediction model, they utilized extreme learning machine (ELM) and integrated PSO algorithm and GWO to control the system’s hyper-parameters. The purpose of combining these two optimizers is to capitalize on the strengths of PSO in exploration and GWO in exploitation. The use of many other metaheuristic optimizers was reviewed in^36^.

Despite several attempts, the use of deep learning for Q and H prediction is limited by three fundamental issues: a shortage of training data, the prevalence of noise and outlier data, and the difficulty of adjusting the model’s hyper-parameters. To this end, this paper proposes a novel deep-learning-based method for dealing with Q and H prediction that can overcome all the abovementioned limitations.

## Proposal

This section introduces three techniques that will be used in our proposed method, namely CNN, LSTM, and SSA.

### Preliminaries

#### One dimensional convolutional neural network (1D CNN)

**Figure 1:**
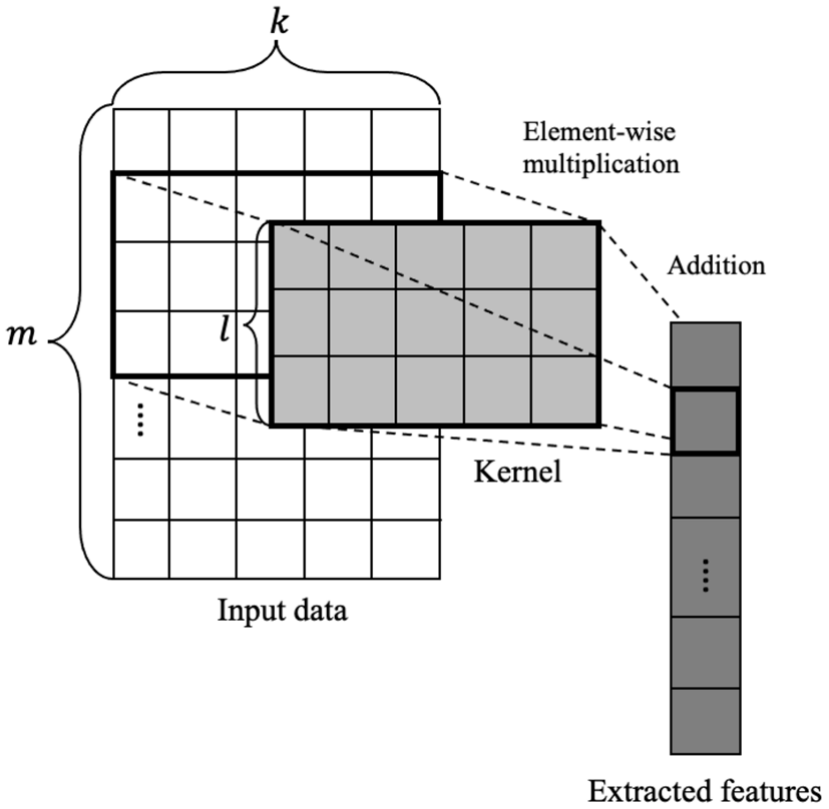
A depiction of the 1-D convolution operation, which employs a kernel of the same width as the input. This kernel performs convolution operations as it traverses the length of the input.

Though CNNs have been widely known for dealing with image tasks, researchers also found its variance’s utility in dealing with time series data. The 1D-CNN model uses a kernel with the same width as the input data. This kernel will traverse through the length of the input data and perform convolution multiplication with the input data’s corresponding component. Specifically, suppose that the input data *x* is a time series with *m* timesteps’ length and *k* features’ width. To get a feature map, we use a kernel $\mathcal {K}$ of width *k*, and execute element-wise multiplication of the kernel and the corresponding portion of the time series *x*. The *i*-th component of the extracted feature map is calculated as follows:

1 $$\begin{aligned} z_i =\sum \limits _{u=i}^{i+l}\sum \limits _{j=0}^{k}x_{uj}*\mathcal {K}_j; ~i = 0, \ldots , m-l, \end{aligned}$$

where $\mathcal {K}$ is a kernel vector of length *l*. In this way, 1D-CNN is supposed to extract short-term temporal correlation inside the data and the relationships across the features. Multiple kernels may be utilized simultaneously to enrich the retrieved information. The training procedure will assist in determining the optimal kernels for extracting the most useful information. Figure 1 illustrates an overview of a CNN model.

**Figure 2:**
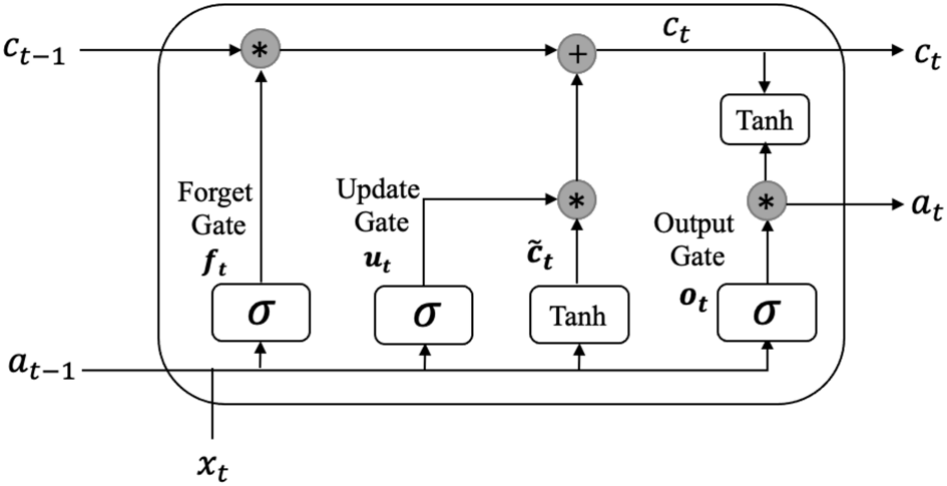
Structure of LSTM cell, which introduces three special gates: Input Gate (i), Forget Gate (f), and Output Gate (o). These gates determine what information to forget, what information to maintain, and how much to store.

#### Long short term memory (LSTM)

As previously mentioned, time series problems require investigating the additional complexity of sequence dependence among input variables. A powerful type of neural network designed for that purpose recurrent neural network (RNN).

Traditional RNNs, on the other hand, encounter two significant challenges when dealing with long sequences: exploding or vanishing gradients. The former describes a situation in which the gradient accumulating via steps gets excessively large, while the latter describes a circumstance in which the gradient drops smaller and smaller, eventually approaching zero. Both of these phenomena might result in the training process being halted or the model failing to converge to the optimal value. To this end, the LSTM network introduces three special gates: Input Gate (i), Forget Gate (f), and Output Gate (o). The LSTM cell may determine what information to forget, what information to maintain, and how much to store by using these gates. In this way, LSTM demonstrates a superior ability to capture long-term dependencies in data. Figure 2 illustrates the structure of a LSTM cell. For each timestep *t*, the formulas to calculate each state are defined as follows:

$$\begin{aligned} c^{~}_{t}&= tanh(W_c[a_{t-1}, x_{t}] + b_c) \\ u_{t}&= \sigma (W_u[a_{t-1}, x_{t}] + b_u) \\ f_{t}&= \sigma (W_f[a_{t-1}, x_{t}] + b_f) \\ o_{t}&= \sigma (W_o[a_{t-1}, x_{t}] + b_o) \\ c_{t}&= u_t * c^{~}_t + f_t * c_{t-1} \\ a_{t}&= o_t * tanh(c_t), \end{aligned}$$

where $x_t$ denotes the input data, $c_t$ depicts the cell state, $c^{~}_{t}$ describes the prospective candidate for memory cell replacement, $u_t$ represents the update gate and $f_t$, and $o_t$ are the outputs of updated forget gate and output gate, *W* denotes the weight matrix; *b* corresponds to the bias coefficient; $\sigma$ and *tanh* are activation functions, respectively.

### Singular-spectrum analysis (SSA)

SSA is a non-parametric method that combines principals of time series analysis, multivariate statistics, dynamical and signal processing. The input data will be split into several components in the SSA approach, each representing a distinct aspect of the original data, such as a trend, oscillations, and noise. SSA is divided into two phases: decomposition and reconstruction. The decomposition phase transforms the input time series into a matrix and then decomposes that matrix into the sum of elementary matrices. Specifically, let ${\textbf {x}}=(x_1, x_2, \dots , x_M)$ be the the input, then, it is divided into a sequence of the so-called *Lagged Vectors*. Each Lagged vector, denoted by $L_i$ consists of *L* elements, i.e., $L_i= {(x_i, \ldots , x_{i+L-1})}^T$, $i=1, \ldots , K= M-L+1$. The Lagged Vectors are then transposed and put together to form a so-called *Trajectory Matrix* $\mathbf {X}$ with the size of $(L \times K)$ as follows,

$$\begin{aligned} \mathbf {X}=\begin{bmatrix} x_1 &{} x_2 &{} x_3 &{} \ldots &{} x_K \\ x_2 &{} x_3 &{} x_4 &{} \ldots &{} x_{K+1} \\ x_3 &{} x_4 &{} x_5 &{} \ldots &{} x_{K+2} \\ \vdots &{} \vdots &{} \vdots &{} \vdots &{} \vdots \\ x_L &{} x_{L+1} &{} x_{L+2} &{} \vdots &{} x_{L+K-1} \end{bmatrix}. \end{aligned}$$

$\mathbf {X}$ is a *Hankel* matrix. This matrix is then decomposed into the sum of *d* elementary matrices, where *d* is the singular value of $\mathbf {X}$ as follows:

$$\begin{aligned} \mathbf {X} = \sum _{i=1}^{d}\mathbf {X}_i = \sum _{i=1}^{d}\sigma _i \mathbf {U}_i \mathbf {V}^{T}_i, \end{aligned}$$

where $\mathbf {U}_i$ and $\mathbf {V}_i$ are the *i*-th left and right singular vectors of $\mathbf {X}$, respectively. The elementary matrices $\mathbf {X_i}$ ($i= 1, \ldots , d$) will then be divided into groups, and the matrices in each group will be combined to generate a resultant matrix as follows:

$$\begin{aligned} \mathbf {X}^{I_i} = \mathbf {X}_{i_1} + \cdots + \mathbf {X}_{i_{k_i}}, \end{aligned}$$

where $I_i = \{i_1, \ldots , i_{k_i}\}$ represents the indices of the *i*-th group. As a consequence, the trajectory matrix can be depicted as the sum of these resultant matrices, as shown below:

$$\begin{aligned} \mathbf {X} = \mathbf {X}^{I_1} + \cdots + \mathbf {X}^{I_m}, \end{aligned}$$

where *m* is the number of resultant matrices. The final stage in SSA is to transform each resultant matrix $\mathbf {X}^{I_i}$ a one-dimensional series of length *M* using the *diagonal averaging* method. By applying the diagonal averaging approach for each matrix $\mathbf {X}_{I_i}$, we yield a time series of length *N*. As a result, the original input time series ${\textbf {x}}$ is decomposed into *m* sub-series: $\mathbf {x} = \mathbf {x}_1 + \cdots + \mathbf {x}_m$.

### Discharge and water level prediction using ensemble learning with GA and SSA assistance

This section proposes a novel discharge and water level prediction model. We first formulate the targeted problem in Section “Problem definition”. We then describe the overview of our approach in Section “Overview of the proposed approach”, and the details of the prediction model in Section “Ensemble learning-based prediction model”. Section “GA-based hyper-parameter optimizer” presents the GA-based hyper-parameter optimizer.

#### Problem definition

The task being investigated here is to predict the future values of water discharge as well as hydrology from the information in the past. Our prediction model, in particular, uses information from the previous *m* timesteps to calculate the Q and H values for the following *n* timesteps. The data is processed in the following manner. We define a sliding window of size $m+n$ that splits the original data into chunks consisting of $m+n$ time steps. The sliding window will run from beginning to end of the original data with the sliding step of 1. Each data chunk’s first *m* time steps will be utilized as the prediction model’s input, while the Q and H values of the following *n* time steps will be used as the label. Below is the mathematical formulation of the targeted problem:

**Input:**

$$x_i, x_{i+1}, \ldots , x_{i+m-1}$$

**Output:**

$$\begin{aligned}&\tilde{y}_{i+m}, \tilde{y}_{i+m+1}, \ldots , \tilde{y}_{i+m+n-1} \\&\quad = \underset{y_{i+m}, y_{i+m+1}, \ldots , y_{i+m+n-1}}{\text {argmax}}p(y_{i+m}, y_{i+m+1}, \ldots , y_{i+m+n-1}|x_i, x_{i+1}, \ldots , x_{i+m-1}), \end{aligned}$$

where $x_i, x_{i+1},\ldots , x_{i+m-1}$ is the input time series, $y_{i+m}, y_{i+m+1}, \ldots , y_{i+m+n-1}$ is a vector representing the discharge (Q) and water level (H) values at the following *n* time steps.

#### Overview of the proposed approach

There are three challenges in dealing with water and discharge level prediction problems. They include (1) the existence of noise in the data; (2) the lack of data; (3) the difficulty in optimizing the model’s hyper-parameters. To cope with the first challenge, we leverage Singular-Spectrum Analysis (SSA) to preprocess the data before feeding to the prediction model. The SSA method aims to decompose a time series into several components with simple structures such as a trend, periodic, and noise. Consequently, we can eliminate the noise from the series and extract relevant information. SSA shows its superiority over other denoising methods since it is a model-free technique independent of the model chosen. SSA can be applied to any data without any assumption, which unleashes the model architecture restriction. Second, we exploit the ensemble learning approach to enhance the training dataset. Specifically, an ensemble learning model includes several base learners and a meta-learner. Intuitively, we use base learners to generate synthetic data to train the meta-learner, which will be used as the predictor. Finally, concerning hyper-parameter optimization, we focus on two types of parameters that are the most important following our observation: the selected elementary matrices in SSA and the number of base learners in the ensemble learning model. We then propose a GA-based mechanism to determine the optimal parameters automatically.

GA is a meta-heuristic inspired by the process of natural selection based on Darwin’s theory of evolution. According to^32^, GA can solve optimization problems difficult for classical methods, particularly those with non-linear, stochastic, non-differentiable, or discontinuous objective functions. The search process in GA begins with a set of individuals (i.e., the initial population), where each individual is a solution to the problem, and a fitness function evaluates the goodness of each individual. By performing GA operations on individuals, we improve their fitness over generations. As a result, after several generations, we arrive at a solution with the best fitness value. Although GA cannot guarantee the best solution, it can provide a suitable solution close to the global optimal in a reasonable amount of time. Indeed, GA has been widely used for tuning hyper-parameters of deep learning models^37–39^. It is worth noting that, in addition to GA, a plethora of meta-heuristic algorithms can aid in discovering a sub-optimal solution^36^. In this work, we do not prioritize selecting the best optimizer. Instead, we propose a framework for addressing the three problems associated with water and discharge level prediction. Instead of our proposed GA-based optimizer, one could use any other optimizer.

**Figure 3:**
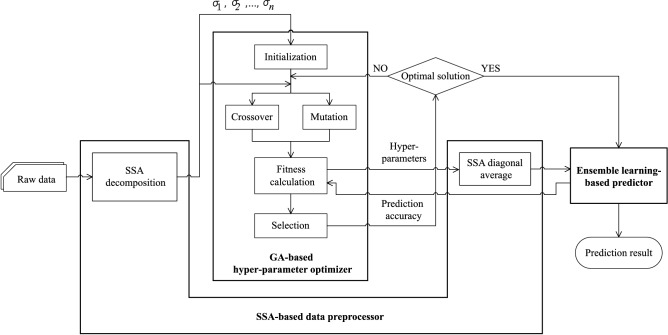
Overview of the proposed model. The SSA-based data preprocessor is responsible for decomposing the raw data into multiple elementary matrices. The GA-based hyper-parameter optimizer determines the sub-optimal parameters for the ensemble learning-based predictor using genetic operations. Finally, the ensemble learning-based predictor forecasts the Q and H values using an ensemble approach.

Figure 3 presents the structure of our proposed model, which has three components: a *SSA-based data pre-processor*, a *GA-based hyper-parameter optimizer*, and a *ensemble learning-based predictor*. These components interact as follows.

- Initially, during a pre-processing phase, the SSA-based data pre-processor decomposes the raw data into multiple elementary matrices, each of which (${\textbf {X}}_i$) is associated with a singular value $\sigma _i$.
- Then, the elementary matrices and their singular values are fed into the GA-based optimizer to determine optimal hyper-parameters of the ensemble learning-based predictor.
- The hyper-parameter optimizer performs the GA operations, including initialization, crossover, mutation, and selection, to search for an optimal solution. A solution’s goodness is evaluated by the fitness value, which is defined by the prediction error when using the solution.
- Once the hyper-parameter optimizer identified the optimal solution, it sends the result to the Ensemble learning-based predictor. The predictor uses the determined hyper-parameters to train the model.

#### SSA-based data preprocessing

As mentioned earlier, the hydrological data usually contains noise and outliers, which may degrade the prediction accuracy. Therefore, eliminating noise and outliers has been considered an essential process that plays a vital role in enhancing the predictive accuracy. To this end, we exploit Singular-Spectrum Analysis (SSA) for removing noise, outliers and filtering out meaningful data before feeding into the prediction model. SSA was chosen because it is a simple but effective strategy that does not rely on model prediction.

The SSA approach begins by converting the input data into a trajectory matrix $\mathbf {X}$, which is then decomposed into a sum of elementary matrices $\mathbf {X_i}$ ($i= 1, \ldots , d$), i.e., $\mathbf {X} = \sum _{i=1}^{d}\mathbf {X_i} = \sum _{i=1}^{d}\sigma _i \mathbf {U}_i \mathbf {V}^{T}_i$. Singular value $\sigma _i$ may be seen intuitively as impact factors that describe the importance of $\mathbf {X}_i$ to the trajectory matrix $\mathbf {X}$. That is to say, elementary matrices $\mathbf {X}_i$ with large $\sigma _i$ will often reflect essential data features such as trend and periodicity. In contrast, elementary matrices $\mathbf { X}_i$ with relatively tiny $\sigma _i$ usually represent noise. Motivated by this fact, our idea is to rely on the value of $\sigma _i$ to eliminate elementary matrices $\mathbf {X}_i$ that are likely to be noise or outliers. In contrast to conventional SSA-based techniques for data processing, we do not manually select $\mathbf {X}_i$. Instead, we employ the GA to determine the sub-optimal combination of $\mathbf {X}_i$ which should be maintained.

Suppose that ${\textbf {x}}$ is the input time series, our proposed SSA-based denoising method is as follows.

- We leverage the *Decomposition stage* of SSA to transfer ${\textbf {x}}$ into the trajectory matrix ${\textbf {X}}$. The trajectory matrix ${\textbf {X}}$ then is represented as the sum of elementary matrices, i.e., ${\textbf {X}} = \sum _{i=1}^{d} {\textbf {X}}_i = \sum _{i=1}^{d}\sigma _i{\textbf {U}}_i{\textbf {V}}^{T}_i$, where $\sigma _i$ are singular values being sorted in the decreasing order.
- The values of $\sigma _i$ are sent to the GA-based hyper-parameter optimizer. The optimizer performs the process described in Section “GA-based hyper-parameter optimizer” to classify the elementary matrices into two groups: the first one presents meaningful information, while the other depicts the noise-liked data. Based on the results received from the GA-based hyper-parameter optimizer, we eliminate the elementary matrices representing noise while remaining the ones depicting meaningful data.
- Let ${\textbf {X}}^*$ the sum of elementary matrices representing meaningful data; We apply the *Diagonal average* method to generates a time series ${\textbf {x}}^*$ from ${\textbf {X}}^*$. ${\textbf {x}}^*$ is the denoised data which will be used to train the prediction model.

#### Ensemble learning-based prediction model

Our ensemble framework consists of several base learners and a meta learner. The base learners have the same structure, including multiple layers of 1D-CNN and LSTM. On the one hand, the use of 1D-CNN layers assists in capturing short-term temporal correlations in historical data. Furthermore, 1D-CNN also helps learn relationships between features in the input data. On the other hand, we can extract the long-term temporal relationship in the data by using LSTM. The primary distinction between base learners is that they will be trained using various epochs. This ensembling mechanism allows us to extract various aspects of the data.

The trained base learners will be used to generate additional training data for the meta learner, which comprises two LSTM layers. The first LSTM layer receives the same input as the base learners. It then extract information from the input and fed to the second LSTM layer. Furthermore, the second LSTM layer receives additional input, which are prediction results given by the base learners. As a result, the second LSTM layer obtains information from many aspects of historical data and will produce high-accuracy predictions. Specifically, the training process is performed as follows.

1. We divide the original data into the three subsets: the training data $\mathbf {x}_{train}$, the evaluation data $\mathbf {x}_{eval}$, and the testing data $\mathbf {x}_{test}$, respectively. $\mathbf {x}_{train}$ and $\mathbf {x}_{eval}$ are pre-processed using our proposed method described in Section “Singular-spectrum analysis (SSA)”, while $\mathbf {x}_{test}$ is left as is.
2. Let $\mathcal {M}_1, \ldots , \mathcal {M}_N$ be the *N* base learners, where *N* is a hyper-parameter that will be automatically adjusted using our proposed algorithm described in Section “GA-based hyper-parameter optimizer”. During the initial training phase, we train all of the base learners with the identical training data $\mathbf {x}_{train}$ but stop at various epochs.
3. We get the trained *N* base models after completing the first step. We’ll start the second training phase, in which we use the trained base learners to produce training data for the meta learner. In particular, we feed the $\mathbf {x}_{eval}$ data into each base learner and get the prediction results. These prediction results are then concatenated to form a synthetic data $\tilde{\mathbf {y}}^{*}$.
4. $\tilde{\mathbf {y}}^{*}$ and $\mathbf {x}_{eval}$ are used to train the meta-learner at the second training stage. To begin, we first send $\mathbf {x}_{eval}$ into the first LSTM layer to extract relevant features. The output of the first LSTM layer is concatenated with $\tilde{\mathbf {y}}^{*}$ to input into the second LSTM layer.

**Figure 4:**
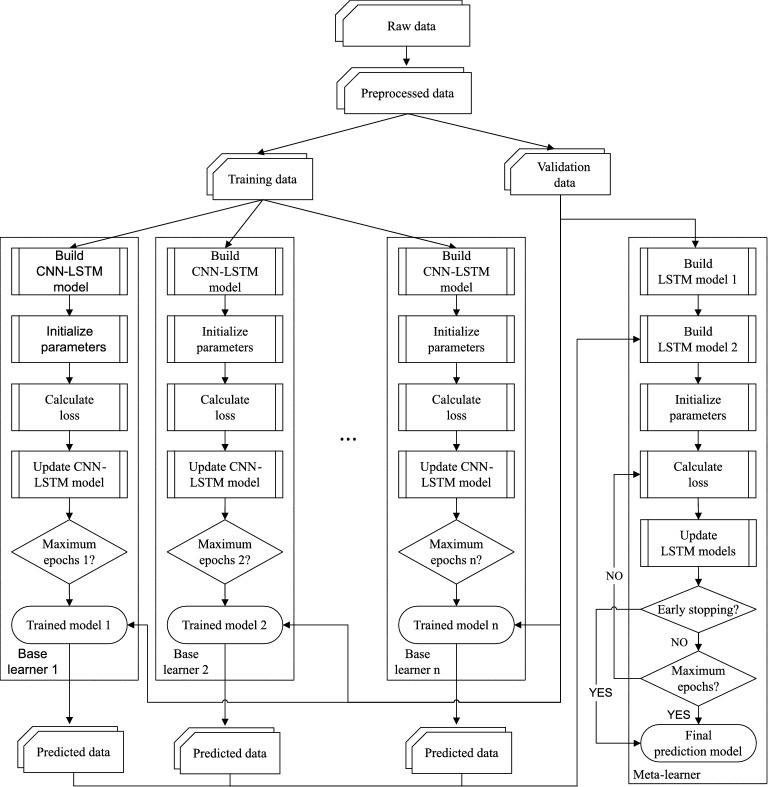
The training flow of the proposed ensemble model. The original dataset is divided into training and validation data subsets. The former is utilized for training multiple base models. The meta-learner is trained using validation data and predicted results produced by the trained base learners.

The training flow is illustrated in Fig.4.

#### GA-based hyper-parameter optimizer

In this section, we propose a GA-based algorithm to optimize the model’s hyper-parameters automatically. The optimization objectives are two-fold. First, we aim at determining a sub-optimal combination of the elementary matrices in the SSA-based denoising task. Second, we want to select the best number of base learners in the ensemble learning-based prediction model. We use a chromosome of $d + 1$ genes, where *d* is the number of elementary matrices. The first *d* genes, which we name *sub-matrices_indicator*, are a sequence of bits 0 and 1, indicating whether the *i*-th elementary matrix is selected. The last gene, named as *base_learner_number*, is a positive integer which represents the number of base learners in the ensemble model. The GA-based hyper-parameter optimizer consists of four main steps, namely, initialization, crossover, mutation, selection. The details of each step will be presented in the next sections.

##### Fitness function and initialization

Initially, we initialize the population by using the traditional approach of randomizing the solutions and our proposed heuristic one. The heuristic initialization algorithm leverages the information from SSA. By using the SSA technique, the adjacent matrix $\mathbf {X}$ is decomposed into the sum of the elementary matrices $\mathbf {X_i}$ ($i= 1, \ldots , d$), i.e., $\mathbf {X} = \sum _{i=1}^{d}\sigma _i \mathbf {U}_i \mathbf {V}^{T}_i$. The singular value $\sigma _i$ represents how important $\mathbf {X}_i$ is with the adjacent matrix $\mathbf {X}$. Thus, a elementary matrix $\mathbf {X}_i$ with a higher $\sigma _i$ is likely to have more impact than a matrix $\mathbf {X}_j$ with a lower $\sigma _j$. From the observation, we design a heuristic initialization algorithm where the elementary matrices with higher singular values will have a higher probability of being chosen. Specifically, the *i*-th gene is set to 1 with a probability of $\frac{\sigma _i}{\sum _{i=j}^{d}\sigma _j}$. We apply the heuristic initialization with a ratio of $p_{i}$ and the random initialization with a ratio of $1-p_{i}$. It is worth noting that the heuristic initialization helps generate good individuals while the random method increases the population’s diversity. The impacts of $p_i$ will be investigated in Section “Performance evaluation”.

The fitness value of an individual is defined by the prediction accuracy of the ensemble model with the hyper-parameters (i.e., the selected elementary matrices and the number of base learners) determined by the individual.

##### Crossover

We apply two crossover schemes for the two parts of the genes, which are the *sub-matrices_indicator* and *base_learner_number*.

Regarding the *sub-matrices_indicator*, we use a combination of two traditional methods: single-point and two-point crossover. In the single-point one, we randomly pick up a so-called crossover point in the two parents’ chromosomes. The genes to the right of the parents then are swapped to result in two offsprings. In the two-point crossover, two crossover points are selected randomly in the parents’ chromosomes. The bits in between of the crossover points are swapped between the two parents.

Regarding the *base_learner_number* part, we leverage a combination of two methods: blend crossover (BLX-$\alpha$) and Flat crossover. The details of these methods are as follows. Let us denote by $n_1$, $n_2$ ($n_1 \le n_2$) the values of the two parents’ genes, and by $n_0$ the value of the offspring obtained by the crossover operation. For the (BLX-$\alpha$) method, $n_0$ is a positive integer selected in the range of $[n_1 - m; n_2 + m]$, where $m = (n_2 - n_1) / 2$. For the Flat crossover, we choose $n_0 = \lambda n_1 + (1- \lambda ) n_2$, where $\lambda$ is a uniform random real number ranges from 0 to 1.

##### Mutation

The mutation operation is designed to generate better individuals while increasing the diversity of the population. To this end, we leverage two methods: multi-point mutation and our proposed heuristic mutation, to mutate the *sub-matrices_indicator* part. For the multi-point mutation, we randomly select two genes in the parents and revert all the bits between these two genes from 0 to 1 and vice versa. Concerning the heuristic mutation, we assign each gene a mutation probability that depends on the corresponding elementary matrix’s singular value. Specifically, if the *i*-th gene has value of 0, its probability for being mutated is $\frac{\sigma _i}{\sum _{j=1}^d \sigma _j}$. In contrast, if the *i*-th gene has value of 1, it will be mutated with a probability of $1-\frac{\sigma _i}{\sum _{j=1}^d \sigma _j}$. The intuition of the heuristic mutation is that genes whose corresponding elementary matrices have high singular values will have more chance for being mutated to 1, and genes whose corresponding elementary matrices have low singular values tend to be mutated to 0. To balance the diversity and goodness of the population, we perform heuristic mutation with a probability of $p_m$, and multi-point mutation with a probability of $1-p_m$, where $p_m$ is a tunable parameter. The impacts of $p_m$ will be discussed in Section “Performance evaluation”.

We apply Gaussian mutation to mutate the *base_learner_number* part. Let $n_1$ be the value of the parent’s *base_learner_number*, then we generate a random number $n_0$ which follows the Gaussian distribution with the mean of $n_1$ and standard deviation of $\sigma$, where $\sigma$ is a tunable parameter.

##### Selection

After performing crossover and mutation operations, we obtain a new population with the size being doubled. We then conduct a selection operation to select appropriate individuals. To this end, we exploit two methods: Tournament selection and Elitism selection. Firstly, we apply Elitism selection to choose individuals with the best fitness values. The portion of individuals chosen by Elitism selection is decided by parameter $p_s$. Then, among the remaining individuals, we apply Tournament selection to choose $50\%-p_s$ of populations. The impacts of $p_s$ will be discussed in Section “Performance evaluation”.

The algorithm terminates when there are more than $50\%$ of individuals who have a performance gap to the best individual less than $10\%$.

## Performance evaluation

This section presents our experimental results for evaluating the performance of the proposed method and comparing it with the existing approaches.

### Study area, datasets and training hyper-parameter configuration

Vietnam, as an agricultural country, is heavily reliant on rivers in a variety of ways. They provide water for various purposes, including irrigation, hydropower generation, land preservation, and drought and flooding prevention. Due to the importance of these functions, a network of hydrological stations has been constructed to monitor their hydrological statistics. In this study, we chose to examine the data from two hydrological stations, Son Tay and Kon Tum, which measure the statistics of two different rivers: the Red River, one of Vietnam’s longest, and the Dakbla River, which flows through several provinces in the country’s south-central highlands. The Red River in Vietnam is 515 km long and has three major branches: the Hong River, the Da River, and the Duong River. Son Tay station, locating at $21^\circ 4.33^\prime$ latitude and $105^\circ \, 30.35^\prime$ longitude in the Son Tay District of Ha Noi, Vietnam, is responsible for measuring the hydrological elements of the Hong River. The hydrological factors are measured daily and hourly during flood seasons using specialized equipment. This study’s dataset is made up of actual measurements taken at this station between January 2008 and December 2019. The second dataset used in this study was collected from the Kontum hydrological station, locating at $14^\circ \,  21.60^\prime$ latitude and $107^\circ \, 59.97^\prime$ longitude in Thang Loi district, Kontum, Vietnam, from January 1985 to December 2015. It measures the same parameters for Dakbla River—a branch of Sesan River, which flows through two provinces Kon Tum and Gia Lai, Vietnam, with the length of main branch of 237 kilometers. These data are transmitted to the National Centre for Hydro-Meteorological Forecasting and are already being utilized in various downstream hydrological activities.

In this study, we focus only on the discharge and water level indices whose max, min, mean, median, and standard deviation (std) are summarized in Table 1.

**Figure 5:**
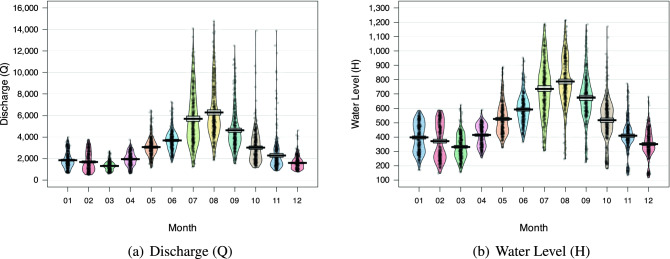
Pirate plot for monthly Discharge and water level of Son Tay dataset. The values during the rainy season (from July to October) are significantly higher than those in the other months.

**Figure 6:**
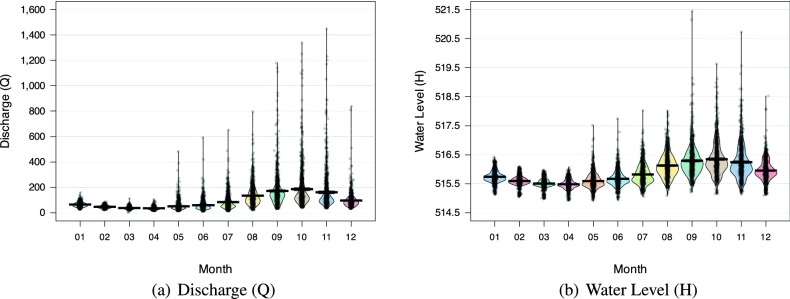
Pirate plot for monthly Discharge and water level of Kon Tum dataset. The mean is relatively small, but the variance is large.

Figure 5 depicts the Discharge and Water levels of the two stations, Son Tay and Kon Tum, respectively. Concerning the former, the distinction between the rainy season (July to October) and the rest is readily apparent. During the rainy season, the mean values of both Discharge and Water levels rise significantly more than during the other months. In addition, $95\%$ Highest Density Intervals (HDI) shows that extremely high statistics occur frequently, i.e., the majority of data points are still located within $95\%$ HDI. In contrast, the dataset obtained at the Kon Tum station reveals a significantly different characteristic. From Fig. 6, it is evident that the rainy season occurs between August and November since the mean values of both Discharge and Water levels are significant during these months. The gap between the months, on the other hand, is much smaller than in the Son Tay dataset. In addition, the superfluous data points in this dataset are much larger than their respective mean and fall outside of the $95\%$ HDI. The experimental results mentioned in Section “Comparison with existing approaches” demonstrate conclusively that these properties make the Kon Tum dataset much more difficult for models to learn.

**Table 1:** Detailed statistics of the two datasets.

Data field/Metrics		Max	Min	Mean	Median	Std
Son Tay dataset	Water level (m)	1216	118	510.34	477	197.74
	Discharge level (m^3^/s)	14800.0	493	3105.64	2550	2148.3
Kon Tum dataset	Water level (m)	521.4	514.9	515.8	515.7	0.4
	Discharge level (m^3^/s)	3500.0	3.5	95.4	65.1	106.2

**Figure 7:**
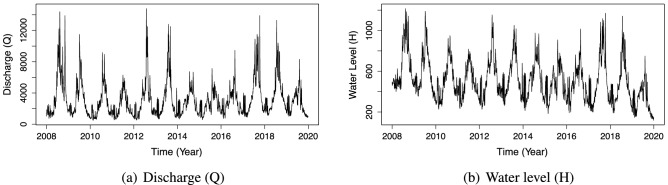
The visualization of the Son Tay dataset’s discharge and water level after removing noise and discontinuous data.

**Figure 8:**
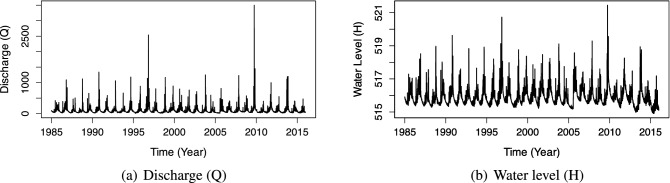
The visualization of the Kon Tum dataset’s discharge and water level after removing noise and discontinuous data.

We see that specific segments have a lot of noise and discontinuous data. Such segments are frequently found at the beginning and end of data sets. As a result, we use the straightforward technique of deleting these portions from the original data. After cleaning the data, we normalize all the values to the range of [0, 1] and divide it into three datasets: the training dataset, validation dataset, and test dataset with a ratio of 0.6, 0.2, and 0.2, respectively. Figures 7 and 8 visualize the discharge values and water level values after cleaning noisy data. We summarize the hyper-parameters of our prediction model in Table 2, where the *iEpochMin* and *iEpochMax* describe the minimum and maximum epoch numbers used in training the base learner, the *iEpochStep* indicates the gap in the number of epochs between two successive base learners.

**Table 2:** The hyper-parameters of the ensemble learning-based prediction model.

Batch size	128	Epoch	200
Optimizer	Adam	Normalizer	MinMax
iEpoch min	100	iEpoch max	250
iEpoch step	50	Patient	100

### Metrics and benchmarks

The prediction models’ performance is evaluated using two types of metrics: error-based and performance-based. The former comprises of four metrics: Mean Square Error (MSE), Mean Absolute Error (MAE), Mean Absolute Percentage Error (MAPE), and Nash-Sutcliffe model efficiency coefficient (NSE) score. The first three metrics measure the error between the prediction results and the ground truth. As a result, the lower MSE/MAE/MAPE, the better the prediction model. NSE, on the other hand, is defined as one minus the ratio of the error variance of the predicted results divided by the variance of the ground truth. Therefore, the greater the NSE, the better the model. Besides the error-based metrics, we utilize the inference time as a performance-based metric. We perform two main experiments. In the former, we evaluate the impacts of the configuration parameters of the GA-based hyper-parameter optimizer. Specifically, we investigate the optimizer’s performance when we vary the settings of GA operations. Based on the experiment results, we provide a guideline in setting the GA-based hyper-parameter optimizer and choose the optimal setting to perform the second experiment. In the second experiment, we investigate the efficacy of the three techniques used in our proposed model: the Ensemble learning approach, SSA-based denoising, and GA-based optimization. We also compare our proposed model with three existing approaches namely ARIMA^40^, ANN^41^, LSTM^18^. Adnan et al. investigated the use of ARMA and ARIMA models for streamflow forecasting in^40^. This work focused on analyzing the characteristics of streamflow data to select the best hyper-parameters. The authors chose the structures of the models after analyzing the plots of ACF and PACF given that preprocessed series. M.Y.A. Khan et al. used a different approach in^41^ to predict discharge and water level. They used multi-layer Artificial Neural Network models (ANN) with three and four layers. The authors used additional input data with relative date-time information to model the seasonal properties of the two factors. The most recent work under consideration is that of Yuli et al.^18^. The authors used a Long Short Term Memory (LSTM) network to predict discharge and water level. They also studied the impact of their method on multi-step predictions. In this study, we also consider two types of predictions: one-step-ahead prediction and multi-step-ahead prediction. In the former, we utilize data from the previous thirty days to forecast the Q and H values for the next day. The latter uses the same input data as the first, but it attempts to forecast the Q and H values many days in advance. Specifically, we vary the number of output time steps from one to seven days. The iterative prediction strategy is utilized, in which the predicted values of the *n*-th day are fed into the model to predict the value of the $(n+1)$-th day.

### Impacts of GA-related parameters

In this section, we investigate the impacts of GA-related parameters including the number of individuals in the population, the ratios for performing heuristic initialization (i.e., $p_i$), heuristic mutation (i.e., $p_m$), and the Elitism selection (i.e., $p_s$).

Although we conducted experiments on both datasets (the Son Tay dataset and the Kon Tum dataset), we present only the Son Tay dataset results in the following to ease the presentation.

#### Number of individuals in a population

In this evaluation, we change the number of individuals between 50, 100, 150 and observe its impacts on the prediction accuracy and the training time. The results are showed in Table 3. As can be observed, using 100 individuals per generation achieves the best performance in terms of prediction accuracy. That is because expanding the number of individuals in a generation helps increase the diversity of the population. Thus, it enlarges the possibility of generating good individuals. However, too many individuals will decrease the ability to combine good parents in the crossover operation. Consequently, there is a tradeoff between the population’s diversity and the convergence speed. The use of 100 individuals balances this tradeoff the best, and thus, it gains the best performance over the other two.

**Figure 9:**
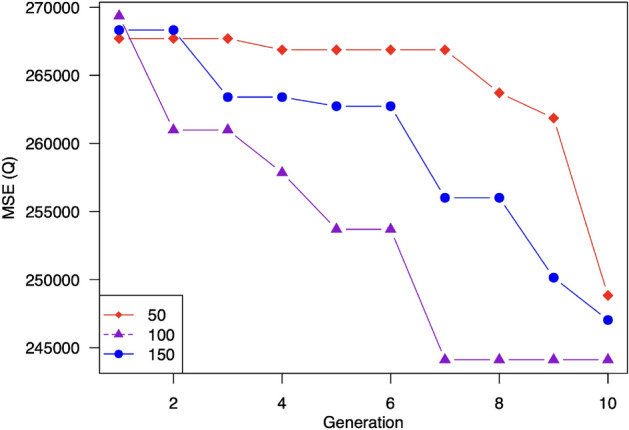
Impacts of number of individuals in population on model performance. MSE tends to decrease when increasing the number of generation. The setting with ten individuals per generation achieves the best performance.

Figure 9 compares the performance of the three settings over the generations. It can be seen that although the setting of 100 individuals has the worst prediction accuracy at the first generation, its performance is improved quickly and outperforms the others from the second generation. The setting of 50 individuals converges the slowest due to too-small search space. Although using 150 individuals improves the performance compared to 50 individuals, its convergence speed is much slower than that of using 100 individuals. As expected, the training time increases proportionally with the number of individuals. Specifically, increasing the number of individuals from 50 to 100 leads to 1.84 times increasing in the training time. Similarly, the gap between training time when using 150 individuals and 100 individuals is 1.75 times.

In summary, we should choose a moderate value of the number of individuals around 100.

**Table 3:** Impact of number of individuals per generation.

Number of individuals	MSE	MAE	MAPE	NSE	Training time
50	1861.03	30.49	10.84	0.914	2550
**100**	**1756.47**	**29.69**	**10.49**	**0.918**	**4712**
150	1920.63	31.38	11.23	0.911	8284

Best results are highlighted in bold text.

The setting with ten individuals per generation achieves the best performance.

#### Heuristic initialization ratio

**Figure 10:**
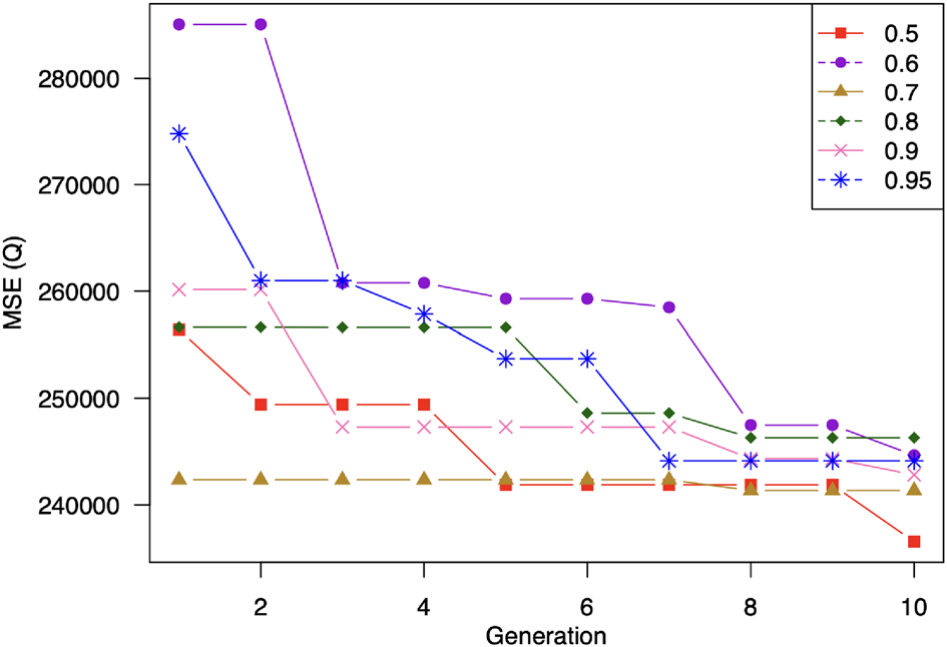
Impacts of percentage of random initialization on model performance. MSE tends to decrease when increasing the number of generation. Using $50\%$ of random initialization achieves the best performance.

We apply a hybrid initialization scheme that combines random and heuristic initialization. The ratios for performing heuristic and random initialization are $p_i$ and $1-p_i$, respectively. We study the impacts of $p_i$ on the performance of our proposal. Specifically, we vary the value of $p_i$ from 0.05 to 0.5 and measure the prediction accuracy and the training time. First, we investigate how much our proposed heuristic initialization algorithm can improve the quality of individuals initialized in the first generation. To do this, we plot the prediction errors corresponding to the best 30 individuals in the first generation in Fig. 10. This figure shows that the prediction error tends to decrease when we increase the value of heuristic initialization ratio, e.g., the prediction errors when $p_i = 0.3; 0.4; 0.5$ are smaller than those of $p_i = 0.05; 0.1; 0.2$. An interesting observation is that using of $p_i = 0.3$ seems to create more good individuals than $p_i = 0.5$. This result is reasonable because an elementary matrix with a higher singular value is not necessarily a more important one. Another interesting result is that although using $p_i = 0.3$ achieves the best quality of the initial population, it causes a low diversity as a trade-off. This drawback narrows the search space and leads to a locally optimal solution as shown in Fig. 10. We can see that the fitness of the best solution over generations with $p_i = 0.3$ is very stable and converges very quickly. In contrast, thanks to balancing the individuals’ diversity and quality, the setting of $p_i = 0.5$ achieves the best performance as shown in Table 4.

Based on the results, we recommend choosing the heuristic initialization ratio of 0.5.

**Table 4:** Impact of heuristic initialization ratio.

Heuristic initialization ratio	MSE	MAE	MAPE	NSE
**0.5**	**1739.46**	**29.3**	**10.16**	**0.9197**
0.4	1888.76	30.26	10.60	0.9128
0.3	1761.33	29.18	10.18	0.9186
0.2	1808.55	28.57	9.36	0.9165
0.1	1957.88	31.1	11.05	0.9096
0.05	1756.47	29.69	10.49	0.9189

Best results are highlighted in bold text.

The heuristic initialization ratio of 0.5 attains the best performance concerning all the metrics.

#### Heuristic mutation ratio

**Figure 11:**
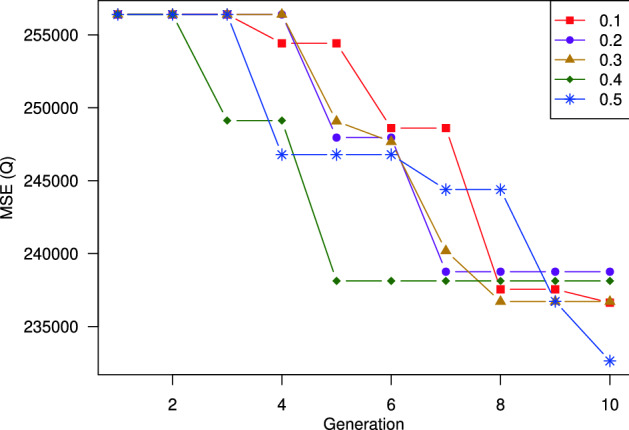
Impacts of percentage of heuristic mutation on model performance. Using the heuristic mutation with the ratio of $0.4\%$ outperforms the others.

This section investigates the impacts of the heuristic mutation ratio $p_m$. We run the experiments with $p_m$ varies from 0.1 to 0.5. Figure 11 depicts the variation of prediction errors over generations, and Table 5 summarizes the results obtained by the best generation of each heuristic mutation ratio. It can be observed that when $p_m$ is small (e.g., $p_m = 0.1, 0.2, 0.3$), the fitness decreases over the generation gradually but the speed is relatively slow. Increasing $p_m$ will speed up the convergence, but it may lead to a local optimum. This phenomenon is reflected clearly in the result of $p_m = 0.4$. As shown, the fitness regarding $p_m = 0.4$ drops severely when moving from the first generation to the third generation and goes horizontally beyond that. The reason can be explained as follows. The elementary matrices with high singular values tend to possess more meaningful data and impact more on the prediction accuracy. By increasing the heuristic mutation ratio, we encourage the genes whose corresponding elementary matrices with high singular values to mutate to 1. In other words, the higher the heuristic mutation ratio, the more elementary matrices with high singular values to be chosen; thus, the more good individuals tend to be generated. Consequently, the fitness will drop quickly. However, the high value of the heuristic mutation ratio leads to a bias on the genes corresponding to the high singular-valued elementary matrices. Therefore, it decreases the diversity of the population and may cause the local optimal phenomenon.

In summary, we should choose the heuristic mutation ratio around 0.4.

**Table 5:** Impact of heuristic mutation probability.

Heuristic mutation ratio	MSE	MAE	MAPE	NSE
0.1	1968.81	31.72	11.44	0.9091
0.2	1851.67	29.67	10.07	0.9145
0.3	1860.54	30.19	10.71	0.9141
**0.4**	**1796.82**	**30.50**	**11.06**	**0.9170**
0.5	1897.73	30.34	10.59	0.9124

Best results are highlighted in bold text.

Using the heuristic mutation probability of 0.4 yields the best performance concerning all metrics.

#### Elitism selection ratio

**Figure 12:**
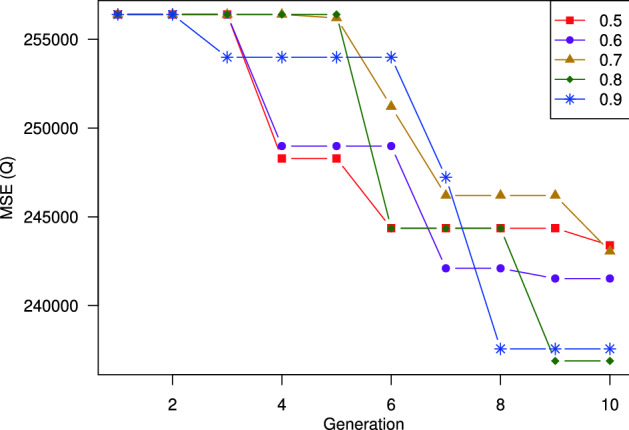
Impacts of selecting best individuals percentage on model performance. Selecting the best individual with the probability of 0.9 get the best performance.

This section investigates the impacts of the Elitism selection ratio, i.e., $p_s$. This parameter shows how many percentages of the best-performed individuals are chosen during the selection process. Figure 12 shows the MSE over the generations when we change the Elitism selection ratio from 0.5 to 0.9. As can be observed, increasing the value of $p_s$ helps improve the solutions’ quality over the generations very quickly. For example, when $p_s = 0.9$, we can see a huge reduction of MSE from generation 6 to generation 8. Meanwhile, with a small value of $p_s$, the prediction error decreases slowly over the generations. The prediction errors concerning all the settings of $p_s$ are shown in Table 6.

According to the results, we recommend setting $p_s$ around 0.9.

**Table 6:** Impact of selecting best individuals of generation probability.

The probability of selecting the best individuals	MSE	MAE	MAPE	NSE
0.5	1897.57	30.36	10.51	0.9124
0.6	1994.14	31.94	11.46	0.9080
0.7	2059.23	32.97	12.42	0.9050
0.8	1905.40	30.92	11.06	0.9120
**0.9**	**1746.21**	**29.31**	**10.18**	**0.9193**

Best results are highlighted in bold text.

Selecting the best individual with the probability of 0.9 get the best performance.

### Comparison with existing approaches

This section evaluate the effectiveness of the three techniques used in our proposed model, namely SSA, Ensemble learning, and GA. Moreover, we compare the model performance with three benchmarks: LSTM, ANN, and ARIMA. Based on the results obtained from the first experiment, the parameters of the GA-based hyper-parameter optimizer are set as follows. The number of individuals in a generation is set to 100; $p_i, p_m$ and $p_s$ are set to 0.5, 0.4 and 0.9, respectively.

#### Impacts of the ensemble learning technique

**Figure 13:**
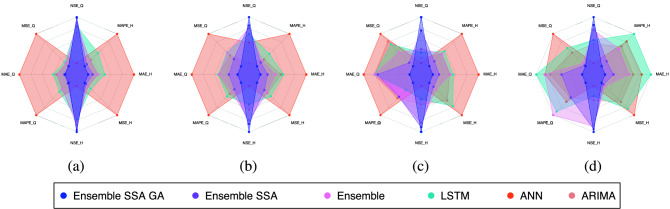
Spider plot indicates all models’ performance. (**a**) Son Tay One-step-ahead (**b**) Son Tay Multi-step-ahead (**c**) Kon Tum One-step-ahead (**d**) Kon Tum Multi-step-ahead. Ensemble SSA GA outperforms the others.

To study the impacts of the Ensemble learning technique, we compare the prediction model using our proposed ensemble learning methodology (without SSA and GA) with the three benchmarks. The experiment results are summarized in Table 7 for the dataset of Son Tay station, and Table 8 for the Kon Tum dataset. To better explain the results, we provide a spider plot in Fig. 13.

As shown, the proposed methods outperform other comparison benchmarks in terms of all the metrics. The Ensemble learning shows huge performance gaps with ANN and ARIMA. Specifically, with the Son Tay dataset, our model increases the NSE factor in Q value prediction by 1.4 and 1.1 times compared to ANN and ARIMA. Regarding H value prediction, the NSE factor is improved by 1.26 and 1.02 times compared to ANN and ARIMA, respectively. Moreover, the MSE, MAE, MAPE attained by the Ensemble model is lower than $2.6\%, 30.4\%$ and $27.5\%$ compared to ANN, in the case of Q value prediction; the gaps in the case of H value prediction are $11.9\%, 31.1\%$, and $27.3\%$, respectively. Our proposed model reduces the MSE, MAE, MAPE respectively by $72.8\%,$ $9.2\%$, and $2.31\%$ concerning Q value prediction, and by $4.85\%,$ $1.1\%$ and $6.99\%$ concerning H value prediction, in comparison with ARIMA. In comparison with LSTM, by ensembling multiple base learners (each combines LSTM and CNN layers), our proposed Ensemble learning methodology improves the NSE by 1.03 times. The MSE, MAE, and MAPE achieved by the Ensemble model are lower than 0.18, 0.88 and 0.78 times concerning Q value prediction, and 0.59, 0.7, and 0.56 times when predicting H value.

Similar results can be captured for the second dataset collected from the Kon Tum station. The Ensemble model enhances the discharge’s NSE scores by $2.83\%$, $12.93\%$, and $7.98\%$, compared with LSTM, ANN, and ARIMA, respectively. Concerning the water level, the performance gaps are $8.15\%$, $13.37\%$, and $6.26\%$, respectively. For other metrics of MSE, MAE, and MAPE, our Ensemble model, in the best case, achieves the result lower by $38.2\%$, $6.92\%$, and $27.6\%$ compared to LSTM, ANN, and ARIMA when predicting Q value. Regarding H prediction, the gaps are $57.14\%$, $28.78\%$, and $29.63\%$.

**Figure 14:**
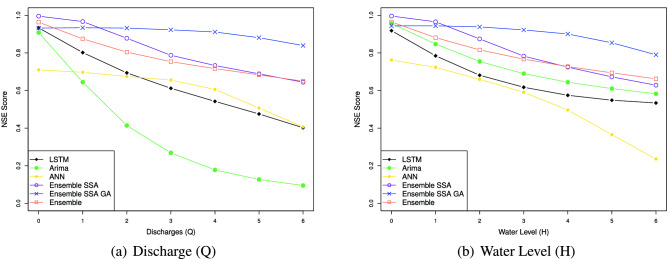
Multi-step-prediction NSE scores of all approaches. The NSE decreases when increasing the number of output timesteps. Overall, our proposed method achieves the highest NSE score.

**Figure 15:**
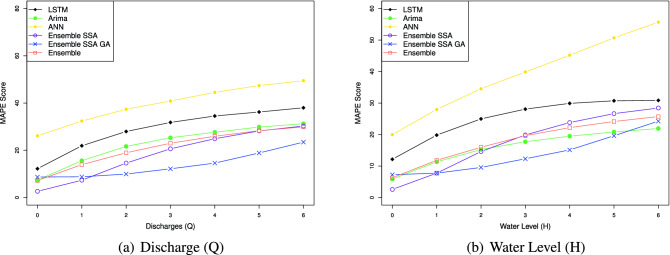
Multi-step-prediction MAPE scores of all approaches. The MAPE increases when increasing the number of output timesteps. Overall, our proposed method achieves the smallest MAPE score.

**Figure 16:**
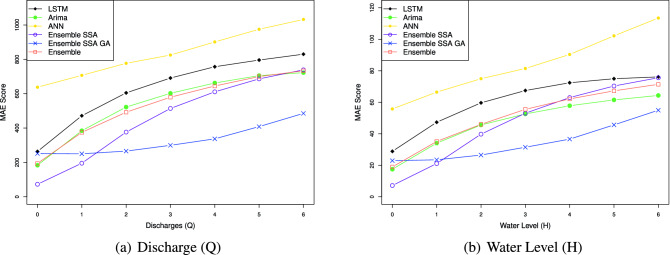
Multi-step-prediction MAE scores of all approaches. The MAE increases when increasing the number of output timesteps. Overall, our proposed method achieves the smallest MAE score.

**Figure 17:**
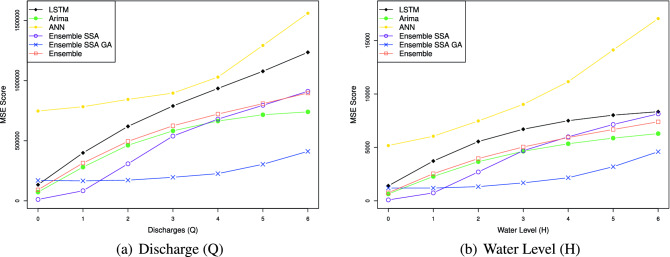
Multi-step-prediction MSE scores of all approaches. The MSE increases when increasing the number of output timesteps. Overall, our proposed method achieves the smallest MSE score.

The efficacy of the Ensemble learning approach is further demonstrated by the multi-steps-ahead prediction results shown in Table 9 for the Son Tay dataset, and Table 10 for the Kon Tum dataset. The table represents NSE, MSE, MAE, and MAPE values when we vary the number of output steps from 1 to 7. Considering the statistics in Table 9, in terms of the NSE factor, the Ensemble model outperforms the LSTM, ANN, and ARIMA by 1.1, 1.3, and 2.2 times, respectively, concerning the Q value; With respect to H value prediction, the performance gaps of the Ensemble model to LSTM, ANN, and ARIMA concerning NSE are 1.2, 1.5, and 1.1, respectively. This result indicates the Ensemble model predicting the future trend better than the others. In terms of the prediction error, the Ensemble model reduces the MSE, MAE, and MAPE by $1.5\%, 2.2\%$ and $14.9\%$ when predicting Q value, and by $16.3\%, 13.7\%$ and $23.5\%$ when predicting H value, compared to LSTM. The performance gap of the Ensemble model to ARIMA ranges from 1.9 and 2.0. The Ensemble model shows a huge gap to the ANN model. Specifically, the Ensemble model reduces the prediction errors by more than $36\%$ compared to ANN in Q and H predictions. Notably, Figs. 14, 15, 16, 17 demonstrate the stability of the Ensemble model in predicting far future. As can be observed, the decreasing slope of the NSE factor achieved by the Ensemble model when increasing the output time steps is the smallest. Similarly, the prediction errors (MAPE, MAE, MSE) caused by the Ensemble model also increase slowest when increasing the output time steps.

The results of the experiment concerning the Kon Tum dataset (Table 10) are comparable to those obtained with the Son Tay dataset. Concerning the discharge prediction, the ensemble model improves the NSE score by at least $18.69\%$ (compared with the LSTM model), and up to $106.5\%$ when being set side by side with ANN. The performance gaps with respect to water level range from $15.62\%$ to $26.52\%$. Regarding the prediction errors, the Ensemble model demonstrates its superiority over the other methods, which is reflected by the significant decrease in MSE, MAE, and MAPE. Specifically, our method reduces the errors by $33.92\%$, $6.85\%$ and $6.77\%$ when predicting Q value, and $40.4\%$, $15.82\%$, $15.79\%$ for H value, compared with LSTM. When putting by the other models, the performance gap is at least $8.57\%$ and up to $46.26\%$ in comparison with ANN, and ranges from $3.51\%$ to $44.05\%$ opposed to ARIMA.

**Table 7:** One-step-ahead forecasting for Son Tay dataset.

Metrics/models		Ensemble SSA GA	Ensemble SSA	Ensemble	LSTM	ANN	ARIMA
Q	NSE	**0.995**	0.99	0.96	0.94	0.71	0.90
	MSE	**11,412**	14,264	76,406	104,972	747,259	72,629
	MAE	**72.92**	85	166	220	637	183
	MAPE	**0.02637**	0.0316	0.0719	0.0919	0.261	0.0736
H	NSE	**0.996**	0.986	0.96	0.93	0.76	0.94
	MSE	**85.62**	288.38	613.7	1041	5173	645
	MAE	**7.17**	11.96	17.31	24.7	55.7	17.5
	MAPE	**0.0258**	0.0366	0.0545	0.0978	0.2	0.0586

Best results are highlighted in bold text.

Our proposed method (i.e., Ensemble SSA GA outperforms the others.

**Table 8:** One-step-ahead Forecasting for Kon Tum dataset.

Metrics/models		Ensemble SSA GA	Ensemble SSA	Ensemble	LSTM	ANN	ARIMA
Q	NSE	**0.871**	0.795	0.690	0.671	0.611	0.639
	MSE	**1821.301**	2238.117	3407.576	4334.260	5522.118	4669.353
	MAE	**16.585**	22.033	22.620	22.568	24.302	22.603
	MAPE	**15.683**	22.252	22.341	18.973	30.875	22.933
H	NSE	**0.943**	0.929	0.916	0.847	0.808	0.862
	MSE	**0.016**	0.021	0.024	0.045	0.056	0.038
	MAE	**0.074**	0.083	0.099	0.103	0.139	0.098
	MAPE	**0.014**	0.016	0.019	0.020	0.027	0.019

Best results are highlighted in bold text.

Our proposed method (i.e., Ensemble SSA GA) outperforms the others.

**Table 9:** Multi-step-ahead forecasting for Son Tay dataset.

Metrics/models		Ensemble SSA GA	Ensemble SSA	Ensemble	LSTM	ANN	ARIMA
Q	NSE	**0.907**	0.81	0.78	0.71	0.61	0.37
	MSE	**235,096**	407,792	564,745	573,311	1,021,919	502,559
	MAE	**328**	424	531	543	836	540
	MAPE	**0.136**	0.189	0.21	0.247	0.397	0.226
H	NSE	**0.89**	0.83	0.80	0.68	0.54	0.72
	MSE	**2193**	3933	4613	5512	10008	4704
	MAE	**34.5**	42.12	50.9	59	83.5	56.6
	MAPE	**0.137**	0.166	0.179	0.234	0.391	0.190

Best results are highlighted in bold text.

Our proposed method (i.e., Ensemble SSA GA) outperforms the others.

**Table 10:** Multi-step-ahead forecasting for Kon Tum dataset.

Metrics/models		Ensemble SSA GA	Ensemble SSA	Ensemble	LSTM	ANN	ARIMA
Q	NSE	**0.611**	0.549	0.508	0.428	0.246	0.396
	MSE	**4746.415**	4919.71	5365.020	8118.964	10,712.340	7251.454
	MAE	**25.738**	32.909	38.824	41.681	38.596	36.815
	MAPE	**21.048**	33.637	48.5814	45.503	37.246	33.725
H	NSE	**0.815**	0.804	0.792	0.667	0.626	0.685
	MSE	**0.053**	0.056	0.059	0.099	0.110	0.087
	MAE	**0.131**	0.143	0.165	0.196	0.183	0.171
	MAPE	**0.025**	0.027	0.032	0.038	0.035	0.033

Best results are highlighted in bold text.

Our proposed method (i.e., Ensemble SSA GA) outperforms the others.

#### Impacts of SSA-based denoising and GA-based hyper-parameter optimization

This section investigates how much the SSA-based denoising and GA-based hyper-parameter optimization can improve the prediction accuracy. To this end, we compare the vanilla Ensemble model with the Ensemble-SSA and Ensemble-SSA-GA models. The difference between the Ensemble-SSA and Ensemble-SSA-GA models is as follows. In the Ensemble-SSA model, we classify the elementary matrices into two groups by their correlation. We then remove the one with lower singular values as it is likely to contain noise-liked data. In the Ensemble-SSA-GA model, the elementary matrices are classified by using the GA-based optimizer. Moreover, the GA-based optimizer is also responsible for determining the optimal number of the base learners in the Ensemble learning-based predictor.

First, the results in Table 7 demonstrate the superiority of Ensemble-SSA and Ensemble-SSA-GA in one-step-ahead prediction performed on the Son Tay dataset. By exploiting the SSA-based denoising method, the Ensemble-SSA model improves the NSE by $2\%$ compared to the model using only the ensemble technique. Concerning the MSE, the Ensemble-SSA model reduces the MAPE by $27.8\%$ when predicting the Q value and $53.0\%$ when predicting the H value. The MAE and MAPE achieved by the Ensemble-SSA model are lower than $44\%$ when predicting Q value, and $69\%$ when predicting H value, compared to the model using only Ensemble technique.

Moreover, using the GA-based hyper-parameter optimizer, the Ensemble-SSA-GA model further increases the NSE and reduces the errors (MSE, MAE, MAPE) compared to the Ensemble-SSA model. Specifically, the performance gaps of NSE, MSE, MAE, and MAPE between the two models are $0.5\%$, $20\%$, $14.2\%$, and $16.5\%$, respectively.

The same experiment is carried out with the Kon Tum dataset to confirm the robustness of our proposed solution. As shown in Table 8, with additional SSA denoising module, the NSE score for Q value is enhanced by $15.21\%$ when comparing with vanilla Ensemble model, and the figure for H value is $2.1\%$. Regarding MSE metric, the Ensemble-SSA model reduces the MSE by $34.3\%$ when predicting the Q value, and $12.5\%$ when predicting the H value. The MAE and MAPE achieved by the Ensemble-SSA model are lower than $2.5\%$ when predicting Q value, and $16.6\%$ when predicting H value, compared to the model using only Ensemble technique. With the process of GA-based hyper-parameter optimization, the performance gaps are even more noticeable. Compared to Ensemble-SSA model, Ensemble-SSA-GA attain the results for NSE, MSE, MAE, MAPE metrics with the average enhancement for Q and H values of $11.29\$$, $3.51\$$, $21.79\$$ and $37.42\$$, respectively.

As can be observed, the Q and H values predicted by the Ensemble-SSA-GA model are very close to the ground truth. Furthermore, it can be observed that the Ensemble-SSA-GA model can forecast the peak of the ground truth data during the rainy season, which is very beneficial for flood prediction applications.

The efficacy of SSA-based denoising and GA-based parameter optimization is further confirmed in the multi-step-ahead prediction. Table 9 summarized the result on Son Tay dataset in this senario. It depicts that by leveraging the SSA technique to pre-process the data, we can increase the NSE and lower the MAPE error by 1.04 times and $34.8\%$ compared to using only the ensemble technique. Moreover, thanks to the GA-based hyper-parameter optimizer, we can improve the NSE by 1.1 times and lower the MSE by more than $40\%$ compared to the Ensemble-SSA model.

Figure 14 represents the models’ NSE factor when varying the output timestep from 1 to 7. When we increase the output time step, we can observe that the NSE of all models drops. An interesting observation is that while the NSE of all the other models decreases significantly when increasing the prediction steps, Ensemble-SSA-GA is relatively stable. As shown in Fig. 14, the NSE of Ensemble-SSA-GA concerning Q prediction is almost unchanged when we vary the output step from 0 to 4, and it decreases by only $9.95\%$ beyond that. In contrast, the NSE of the second-best method (i.e., Ensemble-SSA) decreases by $26.4\%$ when the output step changes from 0 to 4. The stability of the Ensemble-SSA-GA model when increasing output timestep is further proved in Figs. 15, 16 and 17. As shown, the prediction errors caused by Ensemble-SSA-GA increase the slowest among the methods. In detail, the MAPE, MAE, and MSE concerning the 7-days-ahead prediction increase $225\%$, $162\%$, and $177\%$ respectively compared to the 1-day-ahead prediction. On the other hand, these gaps for the second-best method (i.e., Ensemble-SSA) are $1084\%$, $952\%$, and $696\%$, respectively. This result proves the superiority of our proposed Ensemble-SSA-GA model in long-term prediction.

In addition, Table 10 also represent the exceptional performance of our proposed models compared with other methods, conducted on Kon Tum dataset. In term of NSE metrics, Ensemble-SSA-GA and Ensemble-SSA models out-perform the vanilla one by the factor of 1.21 and 1.09 in term of Q prediction, while the gap for H values is $3\$$ and $1.5\%$. For other error metrics, Ensemble-SSA-GA also show its exceptional performance when being compared with Ensemble-SSA and vanilla Ensemble. For the metrics MSE, MAE and MAPE, Ensemble-SSA-GA outperform the vanilla one by $11.52\%$, $33.70\%$ and $56.67\%$ for Q value, and by $10.16\%$, $20.6\%$ and $21.87\%$ for H value. For fast captures of all models’ performance in two prediction senarios and two dataset, we visualize the four Spider plots in Fig. 13. From the plots, the most noticeable insight is that the Ensemble models are all sharing the shape of a slice with a great length in one direction and thin in the other. This shape is the indication for the fact that the two metrics of NSE for Q and H prediction are high, compared to other methods - the lengthy dimension. In addition, the thin side suggests that when putting together with other methods, our proposed Ensemble models achieve a much lower values for all other error metrics (MSE, MAE and MAPE).

#### Inference time

**Table 11:** Mean inference time (in second) of Ensemble models, compared with other methods.

Model/inference time	Ensemble models	LSTM	ANN	ARIMA
	0.109 ($\pm 0.0002$)	0.091 ($\pm 0.0001$)	0.039 ($\pm 0.0002$)	0.026 ($\pm 0.0001$)

In this section, we compare the proposed solution’s inference time to other baselines. The experiment is set up as follows: We pick 100 timings at random and use the methods to predict discharge and water levels at those times. We then record the execution time of each method and summarize it in Table 11 time. The two most straightforward approaches, ANN and ARIMA, take the least amount of time, as expected. However, as demonstrated in previous sections, these two models result in very low prediction accuracy. Although our proposed method requires the highest inference time, the difference between it and LTSM is insignificant. Notably, the time required for one prediction using our proposed method is only 0.109 seconds, which is acceptable in practice.

**Figure 18:**
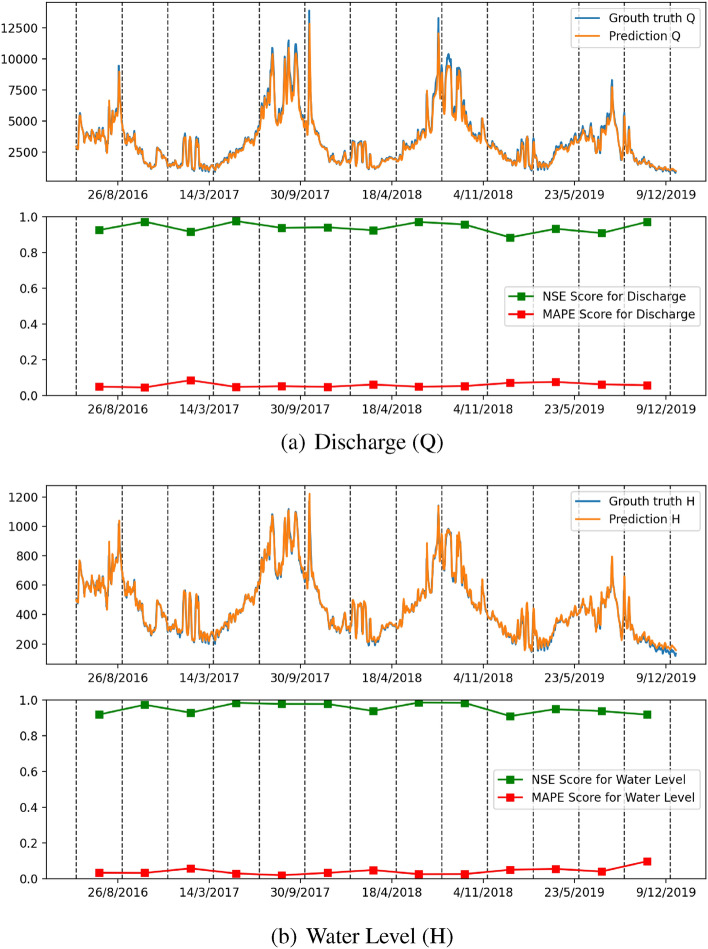
The model performance toward different time segments.

## Discussion

This section will interpret the behavior of our proposed solution under various scenarios. In particular, we first discuss in Section “Dealing with declining trend” our model’s response to the declining trend of two factors: discharge and water level. Then, in Section “Model’s uncertainty”, we present a brief analysis of our model’s uncertainty. Finally, we provide some information about the model’s accuracy.

### Dealing with declining trend

As shown in Figs. 7b and 8b, the amount of water tends to decrease over time. This phenomenon is most recognizable in the Son Tay dataset. There are two factors contributing to this phenomenon. Firstly, the monitoring station locates in the river’s downstream region. Many reservoirs for hydropower and irrigation have recently been built in the upstream regions. A large reservoir in Son La, Lai Chau, in particular, had begun to operate primarily before 2008. As a result, the amount of river water flowing downstream has decreased. Climate change is also having a significant impact on Vietnam. As a result, the amount of rainwater has been decreasing in recent years. Thus, the water level in rivers drops.

Regardless of the data trend, we believe that our proposed solution will function adequately even if the water level drops. This can be explained by the fact that our model is data-driven. As a result, the model will automatically learn the data’s trend and adapt to the change. We use the Son Tay dataset to conduct an experiment to demonstrate this hypothesis as follows. We divide the original dataset into two sub-sets. The data from January 2008 to June 2016 is used to train the model. We then used the trained model to test with the sub-set containing data from June 2016 to January 2020. We measure the average accuracy of the prediction results for every 100-day interval and plot them in Fig. 18. As we can see, the model’s accuracy over all periods has almost no significant change and is entirely unaffected by the average rainfall. Specifically, the model’s accuracy in the segment around August 2017 (when water is the highest) and the model’s accuracy in January 2018 (when water is lowest) is almost identical. Quantitatively, for the former segment, the calculated *NSE* and *MAPE* metrics are 0.937 and 0.051 respectively for the discharge level, while the corresponding values are 0.924 and 0.061 for the latter segment. Taking into account the statistics for water level prediction, the segment corresponding to the highest water level has a score of 0.977 for *NSE*, 0.019 for *MAPE*, compared with the values of 0.938 and 0.048 for lowest water level segment. When taking all segments’ statistics into account, the standard deviation of the *NSE* score is only 0.02 for both discharge and water level, and with *MAPE*, the value is 0.012 for discharge and 0.026 for water level.

### Model’s uncertainty

**Figure 19:**
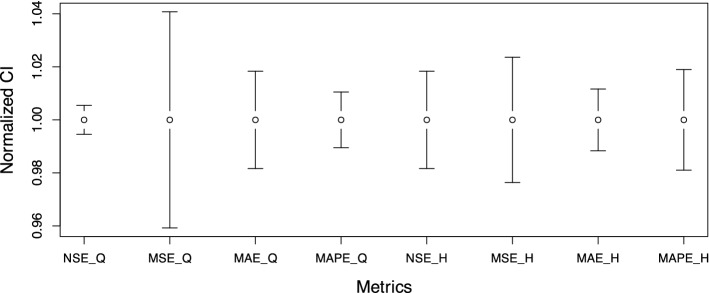
The uncertainty band of each metrics, calculated with the prediction made by Ensemble SSA GA and grouth truth values.

A good hydrological model should possess both characteristics of robustness as well as reliability. Hence, to verify our model’s resilience to changes in input data, as well as the stability of all claimed metrics, we perform an additional experiment for tracking our model’s level of uncertainty. We randomly select 2000 subsets of 500 input sequences from the test dataset (in this case, the Son Tay dataset) and run testing with our proposed model in a one-day-ahead prediction scenario. For each subset, we record the values of metrics *NSE*, *MSE*, *MAE*, and *MAPE*. Following this, we define the uncertainty metric as follows. Let $\mathcal {M}$ be the metrics under investigation; we assume that the value of $\mathcal {M}$ follows a Normal distribution. Under this assumption, we calculate the $95\%$ Confident Interval band of the metrics, $CI_{\mathcal {M}}$, given the sample sets of 2000 data points. The uncertainty, denoted by *d* is defined as the normalized version of CI using the following formula:

2 $$\begin{aligned} d = \frac{CI_{\mathcal {M}}}{\tilde{\mu }_{\mathcal {M}}}, \end{aligned}$$

where $\tilde{\mu }_{\mathcal {M}}$ is the unbiased estimation of the mean of metrics $\mathcal {M}$, produced by our proposed method. The intuition behind this index *d* is that this normalized version of CI is a direct indicator for the level of concentration of a metric toward its mean. The smaller the value of *d*, the lower level of uncertainty. The uncertainty levels of the metrics are shown in Fig. 19. As can be observed, all metrics under investigation achieve the index *d* lower than 0.05, with the lowest value being only 0.003 for NSE scores of Discharge’s prediction, while the highest value is 0.04 for Discharge’s MSE. These numerical results indicate that our model can produce a reliable result on all error-based metrics and is resilient to changes in different input data.

### Model’s accuracy

**Figure 20:**
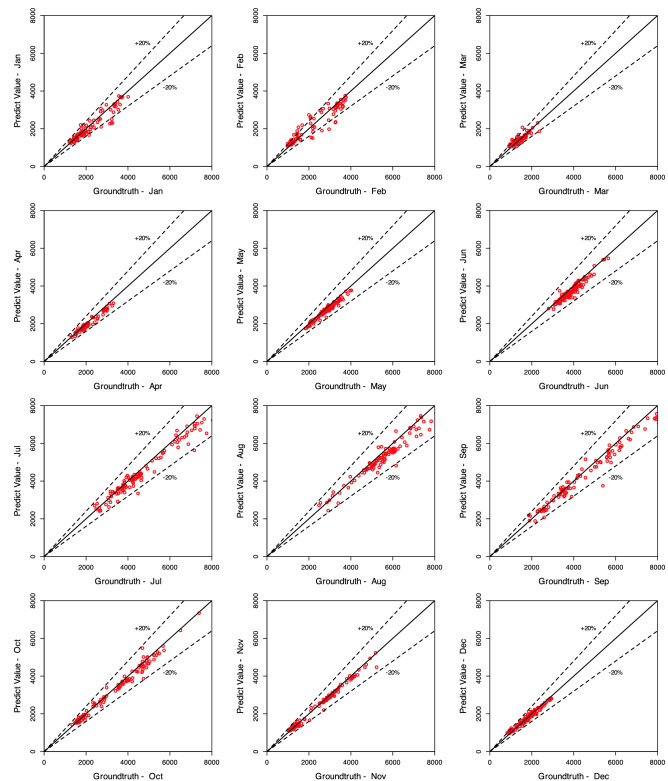
Scatter plot comparing observed and one-step ahead prediction for each month made by Ensemble SSA GA on Son Tay dataset.

In this section, we discuss the proposed model’s prediction results across 12 months in a year. Due to space constraints, we decided to investigate only the one-step-ahead prediction results for the Son Tay dataset’s discharge level from 2016 to 2019. We scatter the ground truth versus the Ensemble SSA GA’s prediction results in Fig. 20). In each plot, there is the best fit line (i.e., the black solid diagonal line) and a $\pm 20\%$ relative error band (i.e., black dashed line). The Ensemble SSA GA model’s forecasts remain within two error bands and are highly concentrated around the best fit line across all of these plots. Notably, the months are divided into two groups. The former involves the flood season, which lasts from July to November, and the latter covers the rest of the year. It can be seen that the value range of the ground truth is quite stable for the group of regular months (not during the flood season), making it more predictable. Furthermore, the model’s predictions for these time intervals are highly consistent and closely match the best-fit line. During the flood season, however, the value ranges of the recorded data are significantly wider, indicating a high frequency of fluctuation in the discharge level. As a result, tracking this rapid shift is more difficult for our model. Although the accuracy is lower during the flood season, it is still adequate and remains within the two $20\%$ relative error areas.

## Conclusion

This work investigates the discharge and water level prediction problem, focusing on two challenges: training data enhancement and hyper-parameter optimization. Concerning the former, we proposed a novel approach that leverages the SSA technique to pre-process the data and the ensemble learning approach to increase the diversity of the training data. To deal with the latter, we presented a GA-based mechanism to determine the optimal combination of sub-matrices and the number of base-learners. We conducted experiments on data acquired from two monitoring stations in Vietnam. The experimental findings demonstrated that our proposed model outperformed the current techniques. In particular, when it comes to one-step-ahead prediction, our ensemble model enhances the NSE score by at least $2\%$ when compared to previous models. Moreover, with the aid of SSA denoising, the performance gap is increased to more than $5\%$. Finally, by leveraging GA-based optimization, we can improve the NSE score by at least $6\%$, and $40\%$ in the best case. Concerning the multi-step-ahead prediction, a similar result is captured. The average NSE achieved by our ensemble model is higher than $9\%$ compared to existing approaches.We further improve the NSE score by $14\%$ with the use of SSA-based denoising and more than $27\%$ by exploiting GA-based optimization.

Despite the fact that the proposed method achieves a high prediction accuracy that significantly outperforms the existing approaches, we are aware of several shortcomings and have plans for future work. To begin, we chose GA to optimize the hyper-parameters in this work. Although GA can produce a suboptimal solution with a short inference time, it requires a significant amount of time to train the model. Moreover, we recognize that many alternative algorithms can meet our requirements. Therefore, our future work will be devoted to determining the most appropriate optimizer. Second, the current scope of this research is limited to the problem of forecasting hydrological parameters (Discharge and Water Level), rather than other types of time series data. In the future, we will apply state-of-the-art techniques such as domain adaptation to transfer the trained model to other domains.

## Data Availability

The datasets used and/or analysed during the current study available from the corresponding author on reasonable request.
